# Evaluating Differences in Whole Blood, Serum, and Urine Screening Tests for Zika Virus, Puerto Rico, USA, 2016

**DOI:** 10.3201/eid2705.203960

**Published:** 2021-05

**Authors:** Asher Y. Rosinger, Samantha M. Olson, Sascha R. Ellington, Janice Perez-Padilla, Regina M. Simeone, Caitlin S. Pedati, Betsy A. Schroeder, Gilberto A. Santiago, Freddy A. Medina, Jorge L. Muñoz-Jordán, Laura E. Adams, Romeo R. Galang, Miguel Valencia-Prado, Sonia Bakkour, Candimar Colón, Mary Goodwin, Dana Meaney-Delman, Jennifer S. Read, Lyle R. Petersen, Denise J. Jamieson, Carmen C. Deseda, Margaret A. Honein, Brenda Rivera-García, Carrie K. Shapiro-Mendoza

**Affiliations:** The Pennsylvania State University, State College, Pennsylvania, USA (A.Y. Rosinger);; Centers for Disease Control and Prevention, Atlanta, Georgia, USA (A.Y. Rosinger, S.M. Olson, S.R. Ellington, R.M. Simeone, C.S. Pedati, B.A. Schroeder, R.R. Galang, M. Goodwin, D. Meaney-Delman, D.J. Jamieson, M.A. Honein, C.K. Shapiro-Mendoza);; G^2^S Corporation, San Antonio, Texas, USA (S.M. Olson);; Centers for Disease Control and Prevention, San Juan, Puerto Rico, USA (J. Perez-Padilla, G.A. Santiago, F.A. Medina, J.L. Muñoz-Jordán, L.E. Adams, C. Colón, J.S. Read);; Iowa Department of Public Health, Des Moines, Iowa, USA (C.S. Pedati);; Pennsylvania Department of Health, Harrisburg, Pennsylvania, USA (B.A. Schroeder);; Puerto Rico Department of Health, San Juan (M. Valencia-Prado, C.C. Deseda, B. Rivera-García);; Vitalant Research Institute, San Francisco, California, USA (S. Bakkour);; University of Vermont Medical Center and Vermont Department of Health, Burlington, Vermont, USA (J.S. Read);; Centers for Disease Control and Prevention, Fort Collins, Colorado, USA (L.R. Petersen);; Emory University School of Medicine, Atlanta (D.J. Jamieson)

**Keywords:** Zika virus, PCR, pregnancy, whole blood, Puerto Rico, outbreak, mosquitos, viruses, zoonoses, vector-borne diseases, mosquito-borne diseases, NAAT, nucleic acid amplification testing, United States

## Abstract

We evaluated nucleic acid amplification testing (NAAT) for Zika virus on whole-blood specimens compared with NAAT on serum and urine specimens among asymptomatic pregnant women during the 2015–2016 Puerto Rico Zika outbreak. Using NAAT, more infections were detected in serum and urine than in whole blood specimens.

Zika virus (ZIKV) infection during pregnancy can cause severe brain and eye malformations and is associated with neurodevelopmental abnormalities in affected infants (*1*). Currently, ZIKV testing with concurrent dengue virus (DENV) testing is recommended for pregnant women who have symptoms and travel to areas with active DENV and risk for ZIKV transmission (*2*–*4*).

Many specimens can be tested for ZIKV, including blood, urine, cerebrospinal fluid, and delivery specimens (e.g., amniotic fluid, placenta) (*1*). Uncertainty still exists about the optimal specimens and tests to detect infection and the duration of detection for each specimen (*4*,*5*). Several reports suggest higher sensitivity of nucleic acid amplification testing (NAAT) on whole-blood and urine specimens compared with serum specimens (*6*–*10*). However, these studies were small or conducted among nonpregnant or symptomatic populations. Since the 2015–2016 Zika outbreak in the Americas, new whole-blood molecular and serologic assays have been approved, but limited data exist on the sensitivity of NAAT for the detection of ZIKV in whole blood specimens among pregnant women. In addition, ZIKV detection might be transient during pregnancy, and absence of a positive test does not indicate lack of infection (*11*). Therefore, we compared ZIKV NAAT results in whole blood specimens to those in serum and urine specimens among asymptomatic pregnant women living in Puerto Rico during the 2015–2016 Zika outbreak.

## The Study

From October 1**–**November 4, 2016, ≈2–3 months after the peak of the Puerto Rico Zika outbreak (*12*), the Puerto Rico Department of Health recruited pregnant women during routine prenatal care visits at 7 clinical sites to provide serum, urine, and whole-blood specimens. Women provided verbal consent, and information was collected on demographic and clinical characteristics. Women with any laboratory evidence of ZIKV infection during pregnancy before recruitment or with any reported clinically compatible symptoms, including fever, rash, headache, eye pain, myalgia, or arthralgia, <7 days before specimen collection were excluded. This study was deemed nonresearch and exempt from institutional review board review.

At collection, specimens were refrigerated at 4°C, transported to the Centers for Disease Control and Prevention (CDC; San Juan, PR, USA) within 12 hours of collection, and stored according to Food and Drug Administration and CDC guidelines (*13*). For each specimen, 200 µL was tested by the Trioplex real-time reverse transcription PCR (rRT-PCR), by using previously described methods (*13*,*14*), including primer pairs specific to ZIKV, DENV, and chikungunya virus (CHIKV). According to surveillance systems in Puerto Rico, DENV and CHIKV circulation were minimal, and no confirmed cases were reported during the study period.

CDC (Fort Collins, CO, USA) performed quality-control testing by singleplex NAAT (*13*,*14*) on all ZIKV-positive specimens. In addition, whole-blood specimens were tested by the Hologic Aptima assay (Hologic, https://www.hologic.com) at Vitalant Research Institute (San Francisco, CA, USA) (*8*). Because results of quality-control testing were consistent and did not change findings, we report Trioplex assay results only.

We also tested serum specimens by the Zika virus IgM capture ELISA (Zika MAC-ELISA) (*15*). We used a positive-to-negative optical density ratio of >3 to determine sample positivity, suggesting previous ZIKV infection (*15*).

Among 514 pregnant women, all were asymptomatic during specimen collection; 14 were symptomatic 8–187 days before collection. The median age was 25 (range 15–43) years; specimen collection was evenly distributed by trimester of pregnancy (Table 1). Of the 1,521 specimens collected, all tested negative for DENV and CHIKV by Trioplex NAAT. Overall, 69 (13%) pregnant women tested positive for ZIKV by NAAT or IgM in >1 specimen (Table 2). A total of 24 (5%) participants tested positive for ZIKV by serum, urine, or whole-blood NAAT and had negative IgM results, whereas 41 (8%) participants had positive IgM and negative NAAT results. Only 4 (1%) participants had positive NAAT and IgM results, and 1 (<1%) woman was positive by NAAT on all specimens and IgM.

**Table 1:** Demographic characteristics among 514 asymptomatic pregnant women, Puerto Rico, USA, October 1–November 4, 2016*

Characteristics	Results
Median age, y (range)	25 (15–43)
Trimester of pregnancy at specimen collection†	
1st trimester: <14 weeks gestation	170 (33)
2nd trimester: 14–27 weeks gestation	187 (36)
3rd trimester: >28 weeks gestation	156 (30)

*Values are no. (%) except as indicated. †Data regarding trimester at time of specimen collection was missing for 1 pregnant woman.

**Table 2:** Number of positive tests for Zika virus among 514 asymptomatic pregnant women tested by specimen type and assay, Puerto Rico, USA, October 1–November 4, 2016*

Category	NAAT			IgM
No. positive tests/no. tested (%)	Median C_t_ value (range)	No. positive tests/no. tested (%)
Specimen type				
Serum	20/509† (4)	37.2 (29.8–37.9)		45/508‡ (9)
Urine	10/503§ (2)	37.4 (36.2–37.9)		
Whole blood	8/507¶ (2)	34.2 (29.8–36.7)		
Total positive tests among pregnant women by test type#	28/514 (5)			45/508 (9)
Total positive tests among pregnant women by any test or specimen type#	69/514 (13)			

*C_t_ values were not compared across specimen types. IgM was performed only on serum specimens. C_t_, cycle threshold; NAAT, nucleic acid amplification testing. †5 serum specimens from asymptomatic women were unable to be provided for Trioplex NAAT. ‡6 serum specimens from asymptomatic women were unable to be provided or were inconclusive for Zika virus IgM testing. §11 urine specimens from asymptomatic women were unable to be provided for Trioplex NAAT. ¶7 whole blood specimens for asymptomatic women were unable to be provided for Trioplex NAAT. #The denominator includes pregnant women who were missing a test type.

Among 28 women who tested positive by NAAT, 8 were by whole blood, 10 by urine, and 20 by serum (Figure). Among the 8 women with NAAT-positive whole-blood specimens, none were positive by only whole blood; 5 tested positive by serum NAAT, 2 by urine NAAT, and 1 by urine and serum NAAT and IgM. Serum NAAT identified 13 positive results not detected by NAAT in another specimen, and urine NAAT identified 6 positive results not otherwise detected (Figure).

**Figure:**
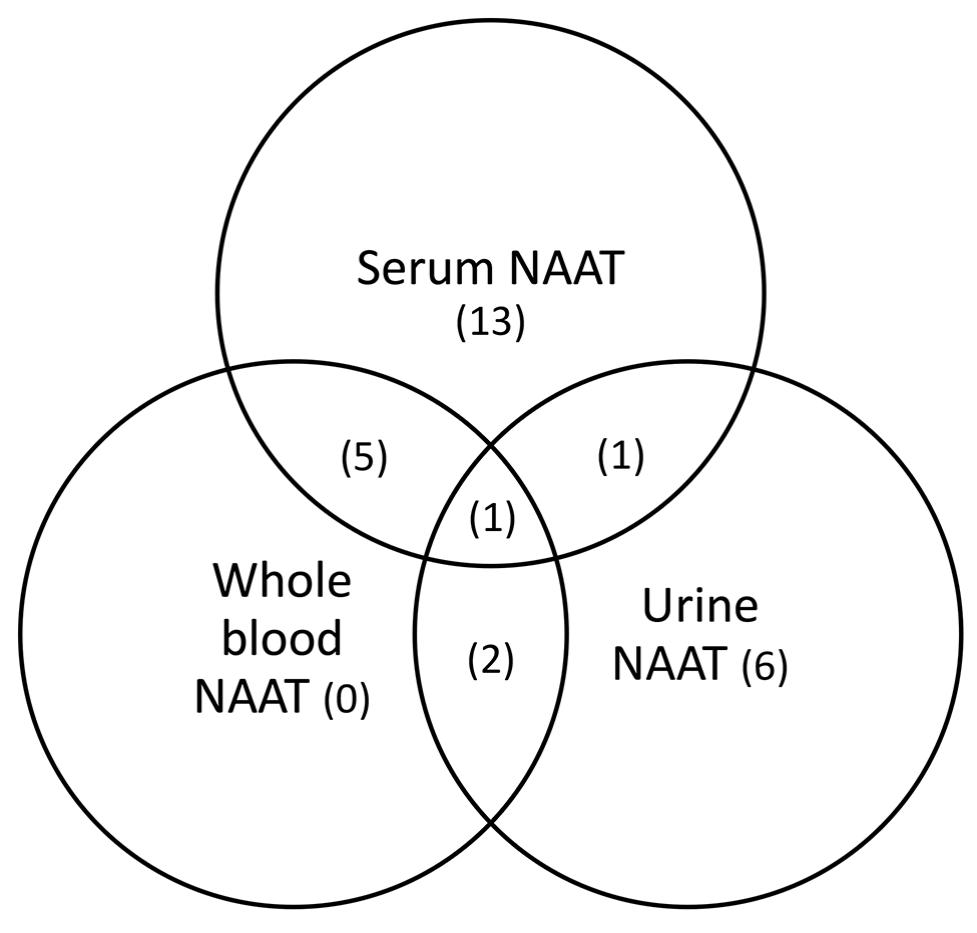
Comparison of positive NAAT results by specimen type for Zika virus infection among asymptomatic pregnant women, Puerto Rico, USA, October 1–November 4, 2016. NAAT, nucleic acid amplification testing.

## Conclusions

This study provides information about laboratory testing to maximize detection of ZIKV infection among asymptomatic pregnant women. In this small sample of ZIKV NAAT–positive asymptomatic pregnant women, no additional ZIKV-positive cases were identified by whole-blood NAAT beyond those identified through tests of other samples. This finding contrasts with other studies that note prolonged detection, higher viral load, and greater sensitivity of whole-blood NAAT versus NAAT on other specimens (*6*,*8*–*10*). Unlike previous studies that tested mostly symptomatic persons (*6*,*10*,*11*), we restricted our analysis to asymptomatic pregnant women.

All asymptomatic ZIKV-positive women had detectable ZIKV in NAAT of urine or serum samples in our study. Although previous studies detected ZIKV RNA in urine more frequently than in serum (*5*,*7*), we found that ZIKV RNA was detected in serum more frequently than in urine; 64% (18/28) tested positive by serum NAAT and negative by urine NAAT. However, serum and urine NAAT together are critical because urine alone identified only 21% (6/28) of pregnant women with a positive NAAT result.

This large study comparing NAAT for ZIKV on serum, urine, and whole-blood specimens is unique in that the study population is among asymptomatic pregnant women. Although studies have mentioned lack of overlap between different specimens tested by NAAT (*7*,*10*) and whole blood yielding fewer positive results in symptomatic persons (*14*), in this study NAAT on whole-blood specimens yielded fewer positive results than NAAT on serum or urine specimens among asymptomatic pregnant women. Because ZIKV-associated birth defects have been noted among asymptomatic pregnant women (*1*), identification of ZIKV infection is critical, especially when the virus is circulating in a community. Timely identification enables appropriate counseling and clinical management.

Our detection of acute ZIKV infections by NAAT is likely low because the study occurred 2–3 months after the peak of the Puerto Rico outbreak (*12*), and false-negative results in pregnant women are possible (*11*). Further, ZIKV RNA–positive results have been reported days or months after symptom onset or first positive test (*6*,*9*), and other cases were reported in Puerto Rico during the study period. Our analysis was among asymptomatic pregnant women, and we could not determine infection onset or whether infection occurred at all. False-positive results were also possible, but samples tested by singleplex NAAT revealed similar results, and results were independently validated in multiple laboratories.

These findings support CDC guidance to perform NAAT on asymptomatic pregnant women during outbreaks when ZIKV is widely circulating (*3*,*4*). Identification of infections among pregnant women who reside in or travel to areas at risk for ZIKV infection is critical for prenatal and postnatal counseling and clinical management (*2*,*4*). Although our understanding of viral persistence in various specimens is growing and the percentage positive in this study was small, NAAT of urine contributed to additional diagnoses, and NAAT on serum and urine combined yielded more positive results compared with whole-blood testing among asymptomatic pregnant women. Timely and accurate prenatal screening and notification of infection can optimize pregnancy and infant care during Zika outbreaks.
